# A novel *small fragment removal system* holds promise to improve stone extraction during lithotripsy

**DOI:** 10.1007/s00345-025-05711-4

**Published:** 2025-06-09

**Authors:** John Lazarus, Mark Wellman, Jeff John

**Affiliations:** 1 Division of Urology, Groote Schuur Hospital, University of Cape Town, Cape Town, South Africa; 2 Department of Urology, Frere Hospital, Walter Sisulu University, East London, South Africa

**Keywords:** Urolithiasis, Retrograde intrarenal surgery, Ureteric access sheath, Intrarenal pressure, Technology

## Abstract

**Objective:**

To describe a novel small fragment removal system (SFRS) which is hypothesized to improve kidney stone fragment removal during laser lithotrypsy. The SFRS consists of three parts: a Syphon Ureteric Access Sheath (SUAS), a Dual Action Pump (DAP) and an Agitator. This clinical trial aims to assess the SFRS’s impact on intra-renal pressure (IRP) and irrigant flow rate compared to a traditional UAS and its stone fragment removal capabilities.

**Materials and methods:**

Patients who met the inclusion criteria were enrolled in this prospective single arm seamless Phase 1 and 2 clinical trial. The primary objective of Phase 1 was to evaluate the safety of the SFRS in terms of IRP changes. In Phase 2 we attempted to evaluate the device’s efficacy in removing stone fragments. During standard laser lithotripsy, the DAP and Syphon UAS were compared to a conventional UAS in terms of irrigant flow and IRP at baseline and during fluid bolus administration. Additionally, the percentage of stone fragments removed using the SFRS with the Agitator introduced (in place of the fURS) was assessed.

**Results:**

Twelve (*n* = 12) patients were enrolled. The SFRS showed lower baseline IRP − 20,8 vs. 24,6mmHg for the traditional UAS (*p* = 0,004). During fluid boluses the IRP was lower for the SFRS at 23,5 vs. 75,2mmHg (*p* = 0,0002). Greater irrigant flow was seen with the SFRS of 39,2 vs. 34,9mL/min (*p* = 0.002). Mean pre-op stone volume was 238mm^3^ (range 70–562 mm^3^), compared to 1,4mm^3^ post-op (range 0–8mm^3^).

**Conclusions:**

The novel SFRS holds clinical promise to improve patient safety by reducing IRP with a significant reduction in IRP during fluid bolus administration. It also has the potential to improve visibility via a significant increase in irrigant flow. Lastly, the SFRS was able to remove the overwhelming majority of stone fragments. The main limitation of the study is the small sample size.

## Introduction

The primary objective of a urological surgeon is to achieve a stone-free status for patients with urolithiasis. Despite breakthroughs in endourological techniques and technology, residual stone fragments persist, placing the patient at risk for subsequent clinical stone episodes. A recent Cochrane meta-analysis on the natural history of fragments following stone surgery revealed an intervention rate of 22% for fragments measuring ≤ 4 mm and 47% for fragments exceeding 4 mm, at 50 months. The disease progression rates were 47% for lesions measuring < 4 mm and 88% for those > 4 mm at 50 months [1]. These findings have led some commentators to assert that the term “clinically insignificant” regarding remaining stone particles post-surgery is likely a misnomer [2].

Contemporary surgical methods employing high frequency laser vaporisation (15–20 Hz) and low energy levels (0.5–1.0 J) can disintegrate stones into the smallest of fragments or “dust.” This raises the question if these < 1 mm fragments can be labelled “insignificant”. The fate of these dusts has been studied by Kang et al. [3]. Their study showed that remnant particles persisted in 60% of patients with dusts. They concluded that any size of post-treatment fragment has the potential to become “clinically significant”. They recommended taking time to basket or wash out dust particles.

To tackle these stone fragments, we developed a small fragment removal system (SFRS). This device is engineered to eliminate small fragments during and immediately after stone fragmentation by using pulsatile and continuous irrigation and suctioning.

We recently described the design of the SFRS and provided the findings of the initial in vitro evaluation to examine its effects on intra-renal pressure (IRP), irrigant flow rate, and stone fragment removal in comparison to a conventional UAS [4].

This paper aims to provide the findings of a human clinical trial of the SFRS, primarily to determine its safety and efficacy. The safety of the SFRS was established if the device did not elevate the IRP over 40 mm Hg. The efficacy of the SFRS is assessed by the proportion of small stone fragments extracted. The SFRS is hypothesised to eliminate over 75% of stone pieces.

## Materials and methods

### Device description

**Fig. 1:**
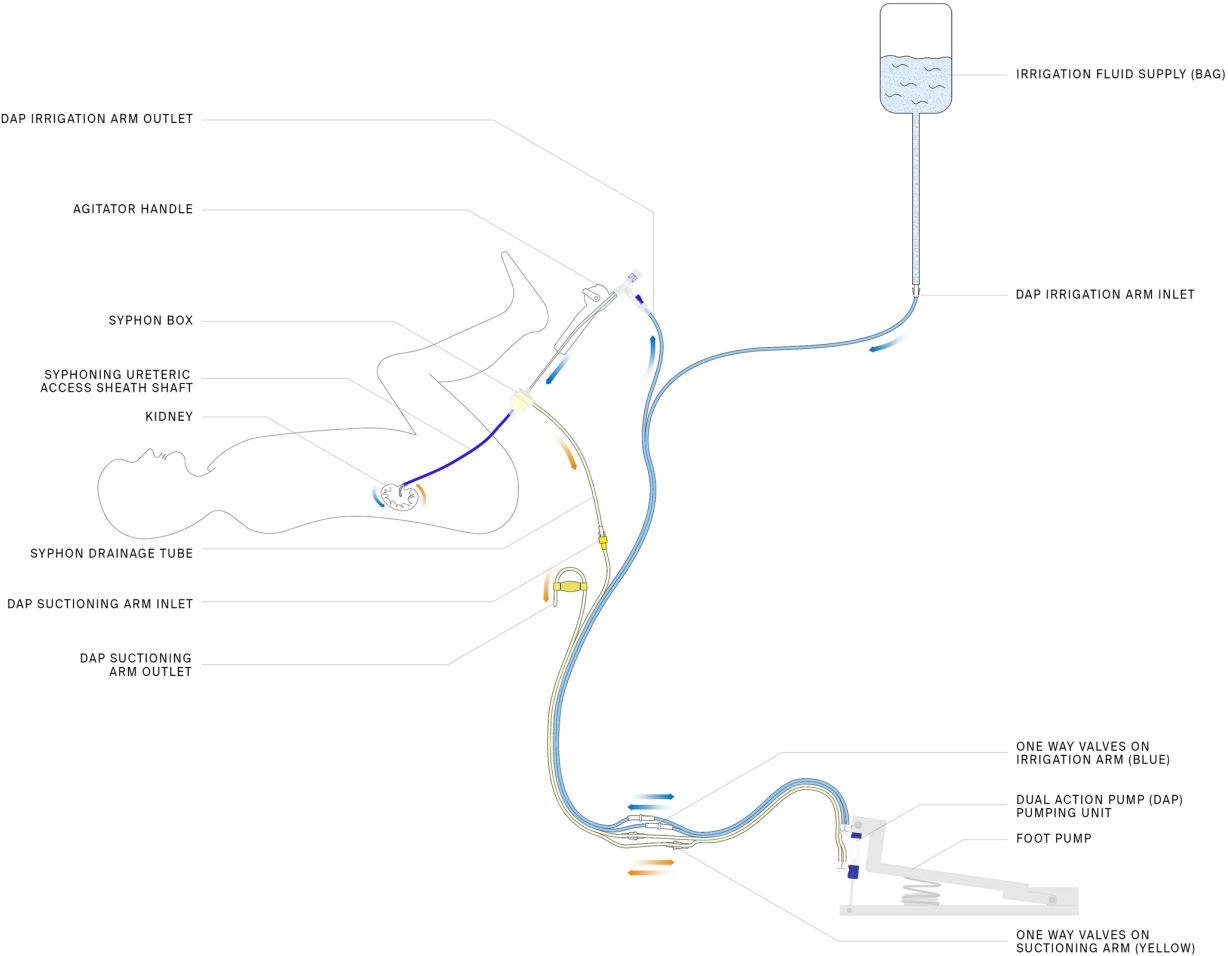
The small fragment removal system (SFRS) schematic showing the different components and how irrigant flows from a suspended bag through a flexible ureterorenoscope to the upper urinary tract. The fluid is then removed via the Syphon UAS. Irrigation and removal of fluid is augmented if the Dual Action Pump (DAP) is activated by the foot pump

**Fig. 2:**
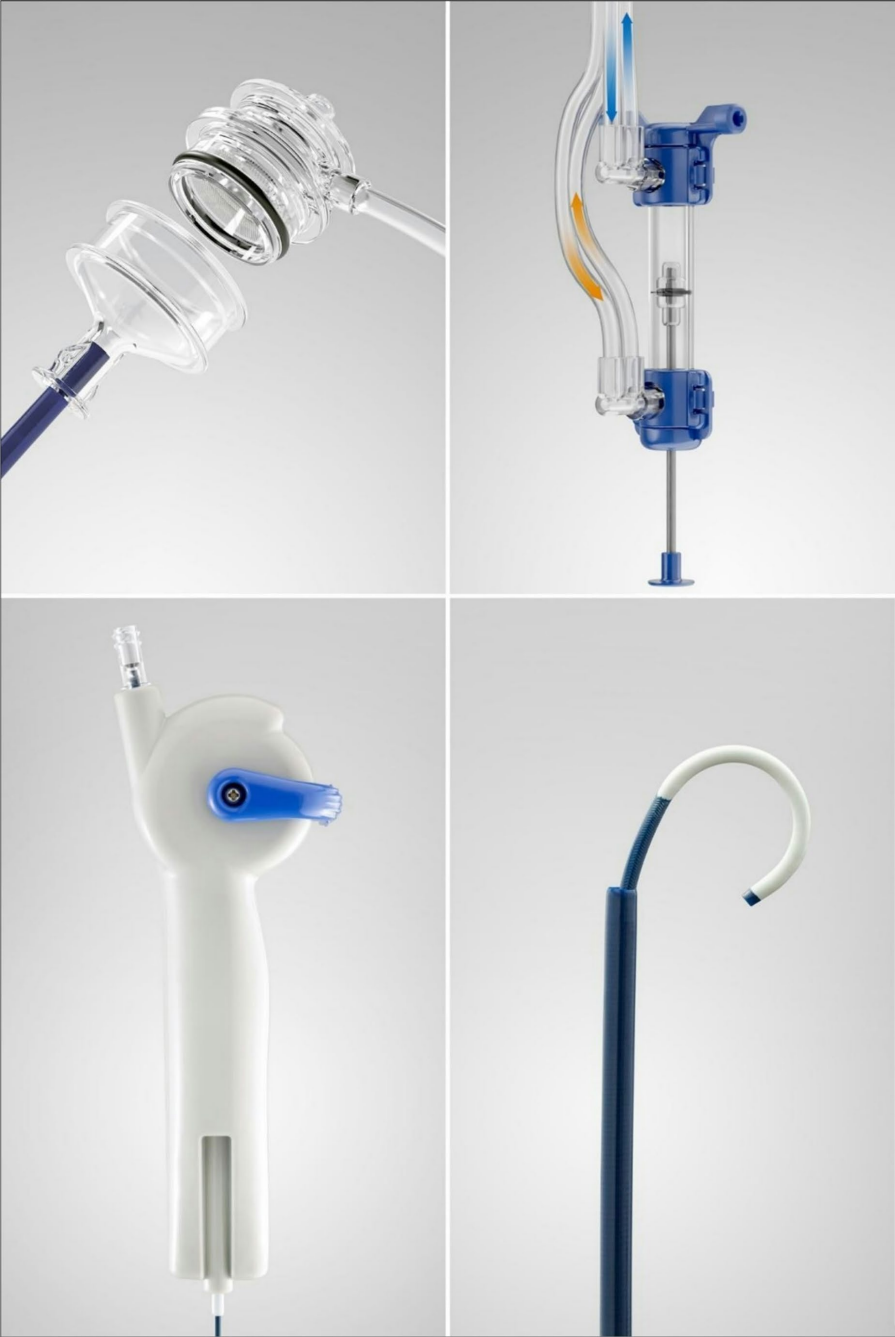
The Syphon Ureteric Access Sheath (upper left), the Dual Action Pump (upper right), Fr 6 Agitator handle with steering mechanism (lower left) and Agitator flexible tip protruding out of the Syphon UAS (lower right)

The SFRS (Figs. 1 and 2) comprises three separate components: the Syphon Ureteric Access Sheath (SAUS), a Dual Action Pump (DAP), and an Agitator incorporated into one system.

The SAUS, previously described by Lazarus [5] and Yekani [6], integrates a syphon mechanism at the outlet of a conventional UAS. This enhances irrigant outflow and diminishing IRP relative to a standard UAS.

**Fig. 3:**
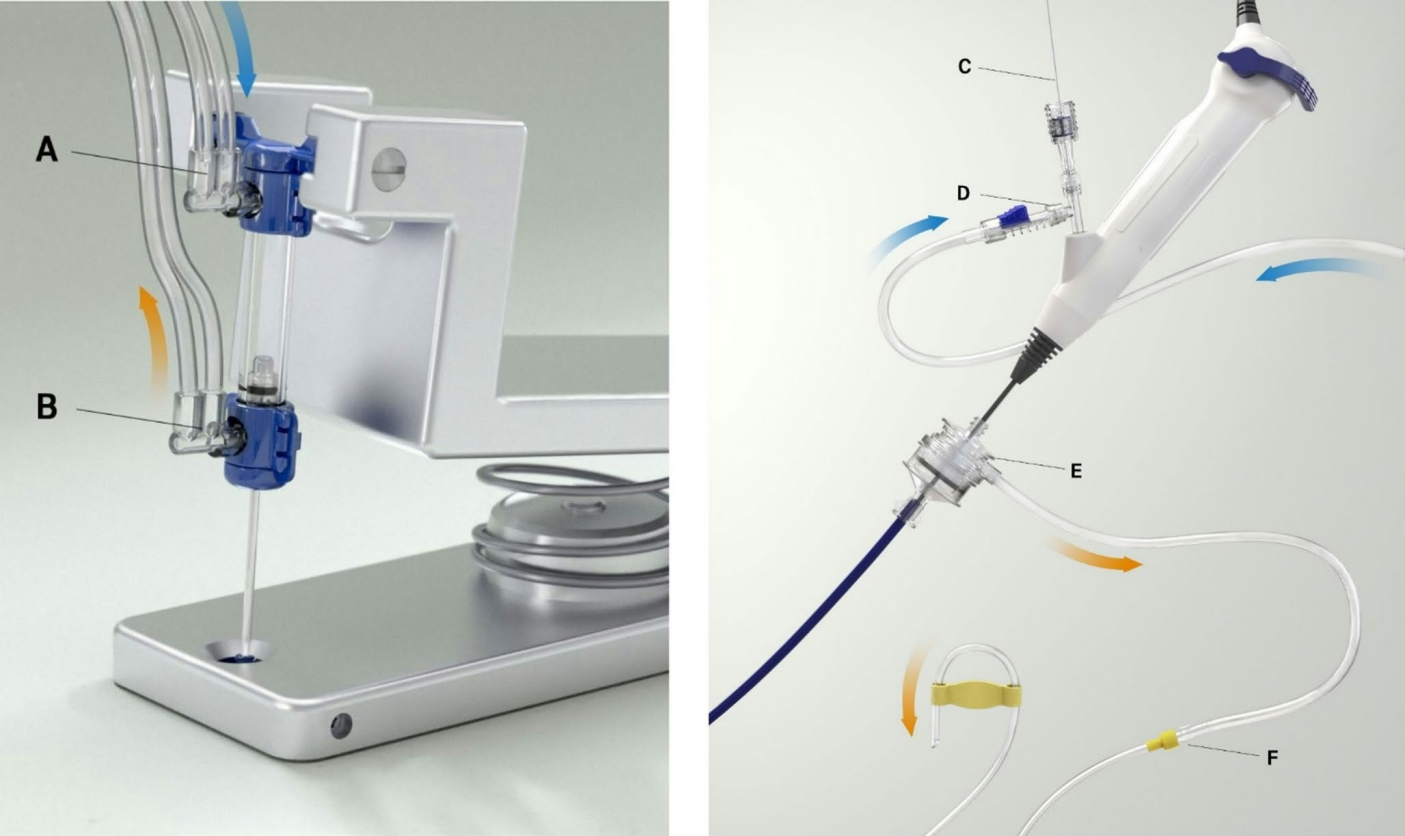
Dual Action Pump (DAP) – foot version (left) with an irrigation arm (**A**) and suctioning arm (**B**). On the right image the SFRS setup is seen with a guidewire (**C**) inserted to standard fURS, an inlet for irrigation (**D**), the syphon UAS (**E**) and the Syphon outlet connected to the suctioning arm of DAP (**F**)

The DAP (Fig. 3) is a low-volume, user-operated (foot or hand) pumping device that allows the urologist to provide fluid boluses of no more than 2mL into the upper urinary tract as the unit is squeezed, simultaneously drawing out the same volume of fluid.

The agitator is a slender (6 Fr) steerable catheter that allows urologists, under fluoroscopic guidance with a radio-opaque marker band, to aim fluid boluses towards stone fragments to facilitate their removal. The Agitator is steered in the same way as a standard flexible ureterorenoscope by moving the thumb lever up and down and rotating the Agitator. At the entrance to each calyx, a few boluses are delivered to make any stone fragments in the calyx waterborne to allow them to be aspirated out, with the action of the DAP.

Stone fragments and dust are retained in a sieve within the Syphon UAS.

**Fig. 4:**
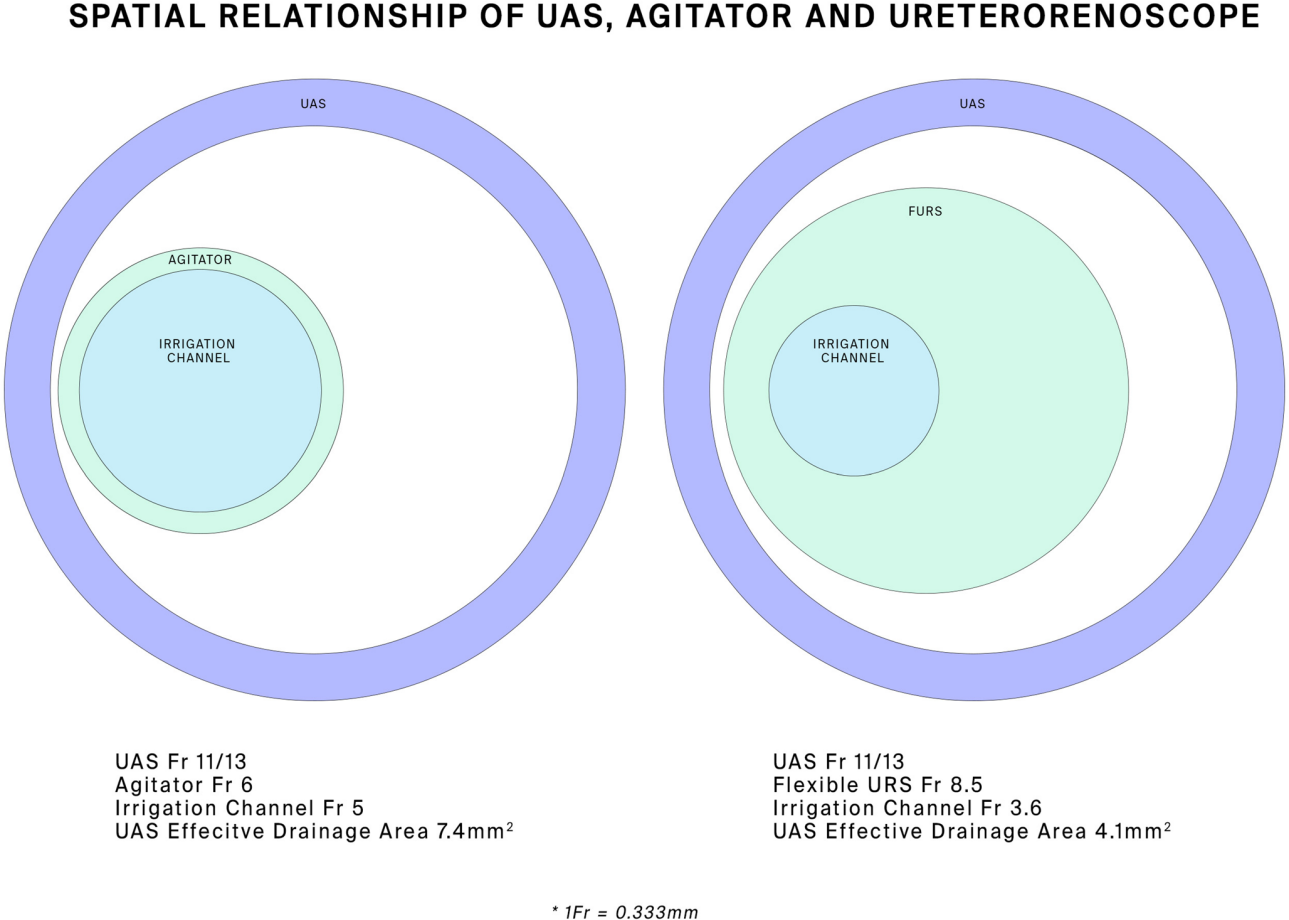
Spatial relationship of ureteric access sheath to the Agitator and a traditional flexible uretero-renoscope

Figure 4 depicts the spatial interaction between the UAS and the Agitator, and a conventional flexible uretero-renoscope.

## Experiment

### Ethical approval

The study received approval from the University of Cape Town’s Human Research Ethics Committee (102/2023). Furthermore, approval was secured from the South African Health Products Regulatory Authority (SAHPRA reference MD20230502), the South African National Clinical Trial Registry (DOH-27-112023-8681) and Groote Schuur Hospital approval (W/SFRS/1).

### Study design

This clinical SFRS trial is a prospective single-arm, seamless Phase 1–2 clinical trial, with an interim data analysis conducted by the Data Safety Monitoring Board (DSMB) between the phases. The primary objective of Phase 1 was to evaluate the safety of the SFRS regarding alterations in IRP. In Phase 2, we sought to evaluate the device’s efficacy in removing stone fragments.

### Endourological procedure

Patients eligible for RIRS who met the inclusion / exclusion criteria provided double consent and were recruited into the trial. Primary inclusion criteria included adults requiring flexible ureteroscopy and laser lithotripsy for a renal stone in the proximal ureter (upper third) or collecting system. Patients were excluded if they had multiple, bilateral or complex stones; if that had a UTI, renal failure of a solitary kidney.

All patients were pre-stented. The ureteric orifice of the affected kidney was cannulated with two guidewires (including one safety wire). Under fluoroscopic guidance, these guidewires were advanced into the collecting system. Thereafter, an 11/13 Fr UAS was inserted over a guidewire with its proximal tip positioned above the pelvic-ureteric junction. (see Fig. 1)

IRP measurements were obtained using a 200 μm fibre optic pressure sensor (FISO Technologies Inc., Quebec City, Canada) calibrated to atmospheric pressure and inserted into the renal pelvis through the UAS. An 8.5Fr flexible uretero-renoscope (WiScope, Series 100, OTU Medical, Union City, CA, USA) was inserted into the upper urinary tract, and a retrograde pyelogram was conducted.

A conventional cysto-irrigation set was connected, with the irrigation bag positioned 140 cm above the kidney level.

Two urological surgeons participated in the study. They had received training on how to use the device. There was no significant learning curve.

### Safety assessment

Three separate tests were conducted to evaluate safety. First, as a control, baseline IRP and minute flow measurements were continuously recorded using the standard UAS. The same variables were measured for the experiment, with the SFRS connected.

Second, the IRP was measured following three slow 2 ml fluid boluses, each administered at 5 s intervals. The maximum IRP (peak pressure) was noted with a conventional UAS, where the DAP was only capable of delivering the irrigant (control) and then with the SFRS (experiment) when the DAP was both delivering irrigant and aspirating from the kidney at the same time.

Similarly, the third assessment involved administering five boluses of the same volume in rapid succession at 1-second intervals. The maximum IRP (peak pressure) was again recorded and noted with a conventional UAS, where the DAP was only capable of delivering the irrigant (control) and then with the SFRS (experiment) when the DAP was both delivering irrigant and aspirating from the kidney at the same time.

### Efficacy assessment

Stone fragmentation was performed with a 20 W laser (Calculase, Karl Storz, Tuttlingen, Germany). The SFRS was then connected, and the Agitator was inserted through the SUAS. Irrigation flow was then started. The DAP was activated, and the Agitator was steered under fluoroscopic guidance to extract as many stone fragments as possible from the collecting system. Short breaks were periodically taken to inspect the collecting system. Following a period of 240 s of DAP activation, all actions were halted.

To determine the efficacy, the stone volume was compared between the pre-operation CT and the post-operation CT performed on the day after the procedure. Volumetric stone analysis was performed using a method described by Preminger et al. [7] The stone volume was calculated using formulas for a prolate ellipsoid for stones < 9 mm maximum diameter and an oblate ellipsoid for stones 9 to 15 mm maximum diameter. The stone volume analysis was performed by a radiologist not allied to the study.

The stone volume clearance rate was calculated as follows:

$$\begin{array}{l}\:Stone\:Volume\:Clearance\:Rate\\=\frac{Residual\:Stone\:Volume\:}{Pre-Operative\:Stone\:Volume}\:x\:100\:\%\end{array}$$

### Data analysis

Statistical analysis was performed using GraphPad Prism version 5.03 software. The paired t-test was used. Statistical significance was regarded as *p* < 0.05.

## Results

Twelve (*n* = 12) patients with renal lithiasis indicated for laser lithotripsy using a flexible uretero-renoscope (f-URS) were enrolled in this study. The mean age was 47.5 years. There were six men and six women.

The baseline IRP with a conventional UAS was 24.6 mmHg, and 20.8 mmHg when the SFRS was attached. The flow rate (minute volume) of conventional UAS was 34.9 mL/min. The flow rate was increased to 39.2 mL/min with the attached SFRS. While administering fluid boluses with the foot pump (three 2mL boluses each given 5 s apart), the maximum IRP increased to 51.6 mmHg when using the conventional UAS. In contrast, the maximum IRP was noted to be 23.6 mmHg when boluses were administered with the attached SFRS. Similarly, when five 2 mL boluses were administered one second apart, the maximum IRP was noted to be 75.2 mmHg and 23.5 mmHg without and with the SFRS attached, respectively (see Table 1). No instances of IRP above the conventionally “safe” 40 mmHg pressure limit were observed for the SFRS.

**Table 1:** The safety comparison results comparing the traditional UAS and the SFRS. *Implies statistically significant (*p* < 0.05)

	Traditional UAS	SFRS	*p*-value
**Flow rate (ml/min)**	34,9	39,2	0,002*
**Baseline Pressure (mmHg)**	24,6	20,8	0,004*
**Peak Pressure (mmHg) during three boluses (2 ml**,** 5 s apart) delivery**	**51**,**6**	**23**,**6**	**0**,001*
**Peak Pressure (mmHg) during five boluses delivery (2 ml**,** 1 s apart)**	**75**,**2**	**23**,**5**	**0**,000*

**Fig. 5:**
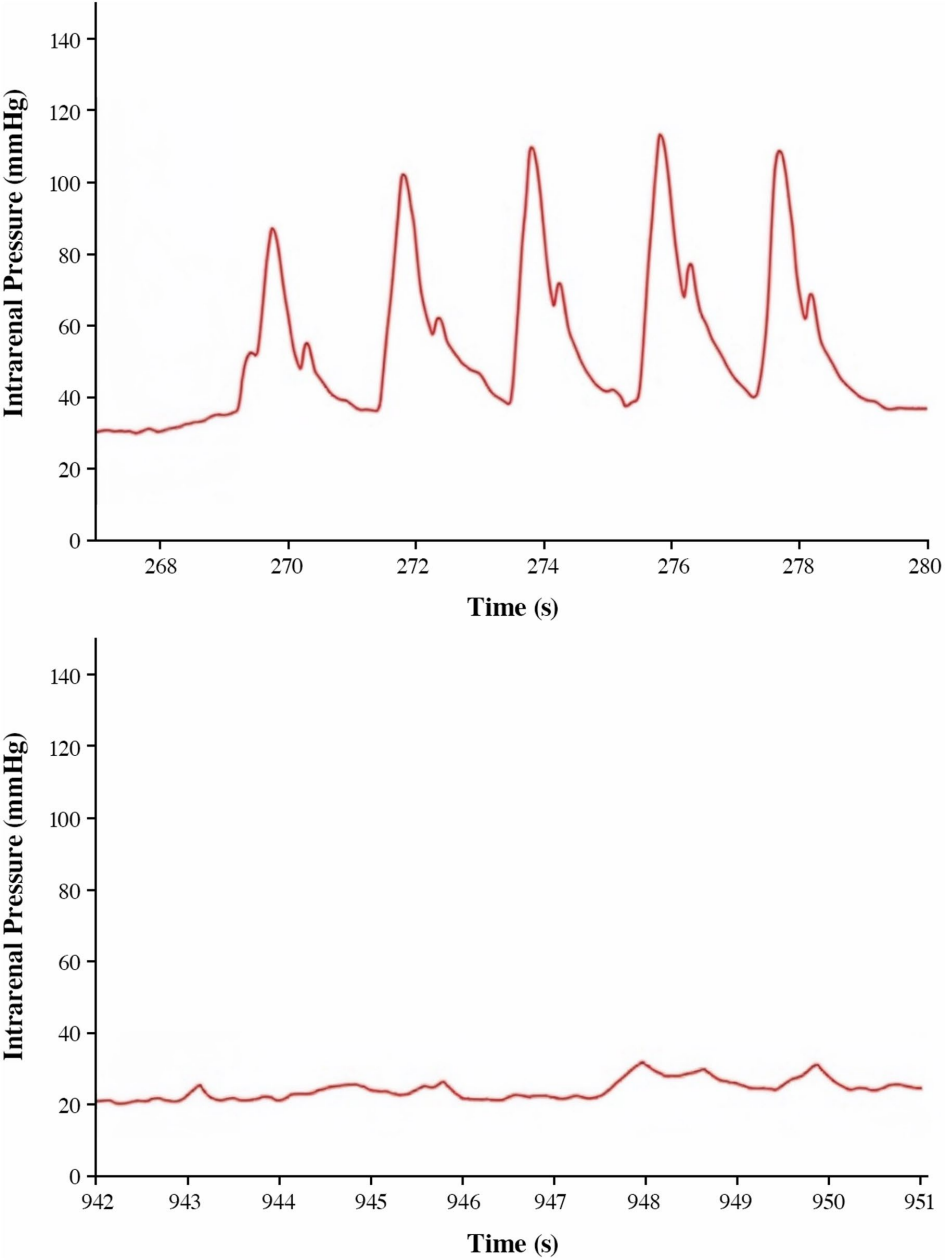
IRP measurements were taken during the fluid bolus assessment. The traditional UAS (left) show a rise in IRP above 100mmHg in this example case, while the SFRS (right) shows stable IRP during periods of vigorous and repeated DAP actuation (five boluses, one second apart). Measurements were taken in the same kidney

Figure 5 displays the IRP readings recorded while administering fluid boluses. For the conventional UAS, the IRP consistently increased from one bolus to the next. However, in the SFRS, the IRP decreased despite periods of vigorous and repeated DAP activation.

The efficacy of the SFRS to remove stones showed a mean pre-op CT scan stone volume of 238mm^3^ (range 70–562 mm^3^), compared to 1,4mm^3^ post-op (range 0–8mm^3^). This represents an average stone clearance rate of 99,3% removal by the SFRS device.

One patient sustained a Post-Ureteroscopic Lesion Scale (PULS) Grade 1 ureteric injury related to the Syphon UAS insertion. This was managed by ureteric DJ stenting. All other patients had an uncomplicated intraoperative course. One patient developed a post-operative urinary tract infection that was managed with oral antibiotics as an outpatient.

## Discussion

Notwithstanding advancements in endourology procedures and technology, achieving a “stone-free” status for patients with urolithiasis continues to pose a problem for endourologists, as a considerable number of patients still retain stone fragments.

A study by Kang et al. showed that 60% of participants with dusts and 75% with residual fragments showed persistence of fragments with 18% and 29% increasing in size respectively during the follow-up period [3]. This led them to conclude that any size of post-treatment fragment may attain “clinical significance” and one should allocate time to either basket or washout dust particles.

The influence of these residual fragments on patients’ quality of life and the associated cost implications merits consideration. Geraghty et al. demonstrated that in the United Kingdom, the national additional cost of treating stone pieces or recurrences is significant. Managing kidney stones equates to an approximate annual expenditure as high as GBP 324 million [8]. This cost is comparable to the combined expenditure on prostate and bladder cancer in UK.

This paper describes a novel SFRS. The combination of the Syphon UAS and DAP with its ability to deliver bolus irrigation while decompressing the kidney by simultaneous aspiration provides a useful method of small fragments removal. Additionally, the very thin diameter (6Fr) Agitator allows the stone fragments to pass through the UAS thanks to the markedly increased effective drainage area as shown in Fig. 4. This clinical trial demonstrated a mean stone clearance rate of 99,3% in the 12 patients studied.

A significant body of recent urological literature has emphasised the importance of maintaining safe IRP and safe working temperatures during laser lithotripsy.

The normal IRP is in the range of 0–15 mmHg (0– 20 cmH_2_O) [9]. fURS is known to increase IRP to 60–100 mmHg in the absence of a UAS. If irrigation is forced, pressure rises of > 300 mmHg are not unusual [10]. In a recent study, Jung and Osther demonstrated in vivo that during routine fURS in 12 patients, the IRP averaged 54 mmHg and pelvic pressure peaks of up to 328 mm Hg occurred. In a 5-min standardised period of simple fURS, 83 pressure peaks of > 50 mmHg were measured [10]. 

Elevated IRP during fURS is a known risk factor for not only urosepsis, but also pain, haemorrhage and acute kidney injury [10]. Zhong et al. studied infective complications and demonstrated in a series of 260 patients undergoing fURS that a systemic inflammatory response was seen in 8.1% [11]. 

In the clinical trial described here the SFRS did not demonstrate any IRP rises above the “safe” limit of 40mmHg. Furthermore, during irrigant bolus administration there was a statistically lower IRP during vigorous continuous application of the Dual Action Pump.

Temperature is also a concern during laser lithotripsy. It is known that cellular thermal injury occurs above 43 °C which can be exceeded even on low-power laser settings [12]. Factors that contribute to elevated intrarenal fluid temperatures include the duration, pulse energy and frequency of laser activation and the flow rate of irrigant fluid [12]. 

The SFRS may mitigate this laser related temperature rise by improving flow rate and enhancing fluid mixing throughout the collecting system of the kidney. This clinical trial showed a significant increase in flow rate of the SFRS compared to a traditional UAS. Additionally, improved flow may also improve visibility for the surgeon. These postulates would need to be evaluated in further trials of the SFRS.

A variety of methods and devices are described in the literature to improve elimination of retained stone fragments. Examples include the following:

1. Various novel stone retrieval basket and grasper designs are described for more efficient stone extraction [13].
2. Devices to prevent ureteral stone retropulsion during intracorporeal lithotripsy such as the “stone cone” [14] or the “backstop” thermosensitive polymer [15].
3. Other investigators have proposed systems to achieve improved aspiration. Du et al. described a perfusion and suctioning platform and modified UAS. That system monitors IRP under negative-pressure suctioning, thus improving flow. They were able to demonstrate improved patient safety by documenting reduced septic marker elevation in those treated with the system [16].
4. Zhu et al. designed a UAS with an additional suction port, which was attached to a vacuum device. They demonstrated improved stone-free rates, reduced infectious complications, and shorter operative time [17].
5. Lastly, a comprehensive review of techniques to minimise fragments by Hein et al. includes other techniques such as advances in laser technology and robotics [18].

Recent studies have underscored the clinical value of suction-enabled ureteral access sheaths and their advanced versions, such as flexible and navigable systems, in optimizing stone clearance, lowering intrarenal pressure, and enhancing intraoperative visibilit [19, 20]

Building on these principles, the SFRS provides constant, gentle suctioning based.

on siphoning effect and introduces an integrated system combining pump, sheath, and agitator, which may offer more precise control over pressure and fragment removal. Further studies will be necessary to evaluate its performance compared to existing devices, especially in more complex stone cases.

## Limitations

This study is not without several limitations. The main limitation is the small number of subjects (*n* = 12) included in this clinical trial. This limited sample size had the potential for patient selection bias for modest stone bulk in favourable intra-renal locations which may limit the results applicability to larger stones or more complex patient anatomy. Postulates about improved intrarenal temperature and improved surgeon visibility consequent on improved irrigant flow were not assessed and would need further validation. Lastly, because the trial was a single arm assessment of the SFRS, the lack of comparative conclusions with a traditional UAS in respect of stone clearance rate needs to be considered.

## Conclusions

The described small fragment removal system is different from traditional UASs by incorporating a syphoning mechanism, a Dual Action Pump which both boluses and simultaneously augments the aspiration of irrigant by the Syphon UAS. It further includes a deflectable Agitator to flush out stone fragments.

In this pilot clinical trial, the novel SFRS holds clinical promise to improve patient safety by reducing IRP with a significant reduction in IRP also during fluid bolus administration. Additionally, it has the potential to improve visibility via a significant increase in irrigant flow. Lastly, the SFRS was able to remove the overwhelming majority of stone fragments. The main limitation of the study is the small sample size, thus these findings need to be verified in a larger trial.

## Data Availability

Data is provided within the manuscript or supplementary information files.

## Abbreviations

fURS: Flexible ureterorenoscopy
IRP: Intra-renal pressure
RIRS: Retrograde intrarenal surgery
UAS: Ureteric access sheath
DAP: Dual action pump
SUAS: Syphon – ureteric access sheath
SFRS: Small fragment removal system
